# *In Vitro* Antimicrobial Activity of Green Synthesized Silver Nanoparticles Against Selected Gram-negative Foodborne Pathogens

**DOI:** 10.3389/fmicb.2018.01555

**Published:** 2018-07-16

**Authors:** Yuet Ying Loo, Yaya Rukayadi, Mahmud-Ab-Rashid Nor-Khaizura, Chee Hao Kuan, Buong Woei Chieng, Mitsuaki Nishibuchi, Son Radu

**Affiliations:** ^1^Department of Food Science, Faculty of Food Science and Technology, Universiti Putra Malaysia, Selangor, Malaysia; ^2^Department of Agricultural and Food Science, Faculty of Science, Universiti Tunku Abdul Rahman, Kampar, Malaysia; ^3^Department of Chemistry, Faculty of Science, Universiti Putra Malaysia, Selangor, Malaysia; ^4^Materials Processing and Technology Laboratory, Institute of Advanced Technology, Universiti Putra Malaysia, Selangor, Malaysia; ^5^Center for Southeast Asian Studies, Kyoto University, Kyoto, Japan; ^6^Laboratory of Food Safety and Food Integrity, Institute of Tropical Agriculture and Food Security (ITAFoS), Universiti Putra Malaysia, Selangor, Malaysia

**Keywords:** silver nanoparticles, tea leave extracts, antimicrobial activity, foodborne pathogens, Gram-negative, time-kill curves

## Abstract

Silver nanoparticles (AgNPs) used in this study were synthesized using pu-erh tea leaves extract with particle size of 4.06 nm. The antibacterial activity of green synthesized AgNPs against a diverse range of Gram-negative foodborne pathogens was determined using disk diffusion method, resazurin microtitre-plate assay (minimum inhibitory concentration, MIC), and minimum bactericidal concentration test (MBC). The MIC and MBC of AgNPs against *Escherichia coli*, *Klebsiella pneumoniae*, *Salmonella* Typhimurium, and *Salmonella* Enteritidis were 7.8, 3.9, 3.9, 3.9 and 7.8, 3.9, 7.8, 3.9 μg/mL, respectively. Time-kill curves were used to evaluate the concentration between MIC and bactericidal activity of AgNPs at concentrations ranging from 0×MIC to 8×MIC. The killing activity of AgNPs was fast acting against all the Gram-negative bacteria tested; the reduction in the number of CFU mL^-1^ was >3 Log_10_ units (99.9%) in 1–2 h. This study indicates that AgNPs exhibit a strong antimicrobial activity and thus might be developed as a new type of antimicrobial agents for the treatment of bacterial infection including multidrug resistant bacterial infection.

## Introduction

Recently, nanotechnology has emerged as a dynamically developing area of scientific interest in the world. Nanoparticles are defined as a nanoscale particle of size ranging from 1 to 100 nm. Among the metallic nanoparticles, silver nanoparticles (AgNPs) have gained increasingly attention due to its unique physical, biological and chemical properties. AgNPs are well-known to exhibit a strong antimicrobial activity against various microorganisms such as bacteria, viruses, and fungi due to its smaller in size and large surface area (Franci et al., 2015). AgNPs are also widely used as anti-fungal (Medda et al., 2015), anti-inflammatory (Hebeish et al., 2014), and anti-viral properties (Bekele et al., 2016).

Green synthesis of AgNPs employing either biological microorganisms or plant extracts has emerged as a simple and alternative to chemical synthesis. Green synthesis method provides advancements over chemical methods as it is environmental friendly and cost effective. Plant extracts-mediated synthesis of AgNPs can be advantageous compared with other biological processes as it does not require the process of maintaining the cell cultures and aseptic environments (Loo et al., 2012). Several studies on the green synthesis of AgNPs using plant extracts have been reported (Medda et al., 2015; Ahmed et al., 2016; Dhand et al., 2016; Selvam et al., 2017).

Foodborne illnesses have emerged as a major public health concern around the world. WHO (2014) reported that there is about 30% of the population in industrialized countries affected by foodborne diseases every year. The consumption of foods contaminated with foodborne pathogens such as bacteria, fungi, viruses, and toxins are often recognized as the main source of foodborne illness in humans. Food especially minimal-processed food can be contaminated during pre-harvesting, post-harvesting, processing, transport, handling, or preparation. The most common foodborne pathogens found in food are *Salmonella* spp. (Lee et al., 2015; D’Ostuni et al., 2016), *Listeria* spp. (Ferreira et al., 2014; Välimaa et al., 2015), *Escherichia coli* O157 (Heiman et al., 2015), *Campylobacter* spp. (Kaakoush et al., 2015), and *Clostridia* spp. (Chukwu et al., 2016).

The presence of multidrug resistance pathogens have increased the number of infectious disease and became the main cause of death in the world (WHO, 2000; Tanwar et al., 2014). Widely misuse and abuse of antibiotics are the leading cause of antibiotic resistance in the bacteria (O’Bryan et al., 2018). Multidrug resistant bacteria infection may lead to several impacts including increase of mortality and morbidity rates, prolong of hospitalization period, and economic loss (Patel et al., 2008). Woh et al. (2017) detected multi-drug resistant non-typhoidal *Salmonella* among migrant food handlers, which may cause cross-contamination to the food products. Thus, the development of a new and natural antimicrobial agent is needed as there is a growing concern in multidrug resistant foodborne pathogens.

The aim of this study is to determine the antibacterial activity of green synthesized AgNPs against a diverse range of Gram-negative foodborne pathogens by using disk diffusion method, resazurin microtitre-plate assay minimum inhibitory concentration (MIC), minimum bactericidal concentration test (MBC), and time-kill curve assay.

## Materials and Methods

### Preparation of Silver Nanoparticles

The synthesis of AgNPs using pu-erh tea leaves extracts was done using the method as described previously (Loo et al., 2012). Ten gram of tea leaves was weighed in a beaker. The tea leaves was added with 100 mL of distilled water and maintained at 60°C for 10 min. After 10 min, the tea extract was filtered using 0.45 μm Millipore membrane filter and followed by 0.2 μm Millipore membrane filter. For synthesis of AgNPs, 12 mL of tea extracts was added into 100 mL of AgNO_3_ (1 mM) in Erlenmeyer flask at room temperature. Color changes of the solution were observed. The synthesized AgNPs were characterized by UV-vis spectroscopy, X-ray diffraction (XRD), Fourier transform infrared (FTIR) spectroscopy, and transmission electron microscopy (TEM).

### Bacteria Strains Preparation

*Escherichia coli* ATCC 25922 (*E. coli*), *Klebsiella pneumoniae* ATCC 13773 (*K. pneumoniae*), *Salmonella* Typhimurium ATCC 14028 (*S.* Typhimurium), and *Salmonella* Enteritidis ATCC 13076 (*S.* Enteritidis) were obtained from the American Type Culture Collection (Rockville, MD, United States). All the bacteria strains were cultured in Mueller Hinton broth (MHB) (Merck, Germany) at 37°C for 24 h with 200 rpm agitation.

### Preparation of Resazurin Solution

The resazurin solution was prepared at 0.02% (wt/vol) according to Khalifa et al. (2013). A 0.002 g of resazurin salt powder was dissolved in 10 mL of distilled water and vortexed. The mixture was filtered by Millipore membrane filter (0.2 μm). The resazurin solution can be kept at 4°C for 2 weeks.

### *In Vitro* Susceptibility Test

#### Disk Diffusion Method

The antibacterial activity of AgNPs against the selected Gram-negative foodborne pathogens was carried out using Kirby–Bauer Disk Diffusion Susceptibility Test method (Bauer et al., 1966). The bacteria strains were spread on the Mueller-Hinton agar (MHA) (Merck, Germany) using sterile cotton swab. Sterile blank antimicrobial susceptibility disk was used in the test. The disks were loaded with 10 μL of tea leaves extracts, silver nitrate solution (1 mM), and solution containing tea leaves mediated synthesized AgNPs separately. The disks were then placed on the agar plate and incubated at 37°C for 24 h. The zone of inhibition was observed after 24 h of incubation.

#### Minimum Inhibitory Concentration (MIC) and Minimum Bactericidal Concentration (MBC) Evaluation

The MIC and MBC of green synthesized AgNPs were done using the method described in the guideline of CLSI (2012). The MIC test was performed in 96-well round bottom microtiter plate using standard broth microdilution methods while the MBC test was performed on the MHA plates. The bacterial inoculums were adjusted to the concentration of 10^6^ CFU/mL. For the MIC test, 100 μL of the synthesized AgNPs stock solution (500 μg/mL) was added and diluted twofold with the bacterial inoculums in 100 μL of MHB started from column 12 to column 3. Column 12 of the microtiter plate contained the highest concentration of AgNPs, while column 3 contained the lowest concentration. Column 1 served as negative control (only medium) and the column 2 served as positive control (medium and bacterial inoculums). Each well of the microtiter plate was added with 30 μL of the resazurin solution and incubated at 37°C for 24 h. Any color changes were observed. Blue/purple color indicated no bacterial growth while pink/colorless indicated bacterial growth. The MIC value was taken at the lowest concentration of antibacterial agents that inhibits the growth of bacteria (color remained in blue).

The MBC was defined as the lowest concentration of the antibacterial agents that completely kill the bacteria. MBC test was performed by plating the suspension from each well of microtiter plates into MHA plate. The plates were incubated at 37°C for 24 h. The lowest concentration with no visible growths on the MHA plate was taken as MBC value.

#### Time-Kill Curve

Time-kill assay was done in MHB medium as described by Zainin et al. (2013) and Lau et al. (2014). The bacterial inoculums were adjusted to 10^6^ CFU/mL. The AgNPs solution was diluted with MHB media containing bacterial inoculums to obtain the final concentration of 0× MIC, 0.5× MIC, 1× MIC, 2× MIC, 4× MIC, and 8× MIC for each type of bacteria in the total final volume of 1 mL. The cultures were then incubated at 37°C with 150 rpm agitation. The cultures (100 μL) were spread on MHA plates at time 0, 0.25, 0.5, 1, 2, and 4 h. The experiment was carried out in triplicate. The number of colonies on the MHA plates was quantified in CFU/mL after incubation at 37°C for 24 h. For statistical analysis, SPSS (v.19) statistical package was used to determine the significant (*P* < 0.05) difference among the tested foodborne pathogens.

## Results and Discussion

This study was aimed to determine the antibacterial effect of green synthesized AgNPs. The green synthesized AgNPs used in this study were characterized by UV-vis spectroscopy, XRD, FTIR spectroscopy, and TEM. The XRD patterns for synthesized AgNPs showed that five main characteristic diffraction peaks for Ag were observed at 2θ = 38.4, 44.5, 64.8, 77.7, and 81.7, which correspond to the (111), (200), (220), (311), and (222) crystallographic planes of face-centered cubic (fcc) Ag crystals. The UV-vis absorption spectrum of the synthesized AgNPs showed a broad peak at 436 nm which is a characteristic band for Ag. Three infrared bands were observed at 3,271, 1,637, and 386 cm^-1^ in FTIR measurement. The band at 3,271 and 1,637 cm^-1^ indicated that the presence of protein as capping agent for AgNPs which increases the stability of the nanoparticles, while the broad peak at 386 cm^-1^ corresponded to the Ag metal. TEM image revealed that the AgNPs is spherical with the particle size of 4.06 nm (Loo et al., 2012).

**FIGURE 1:**
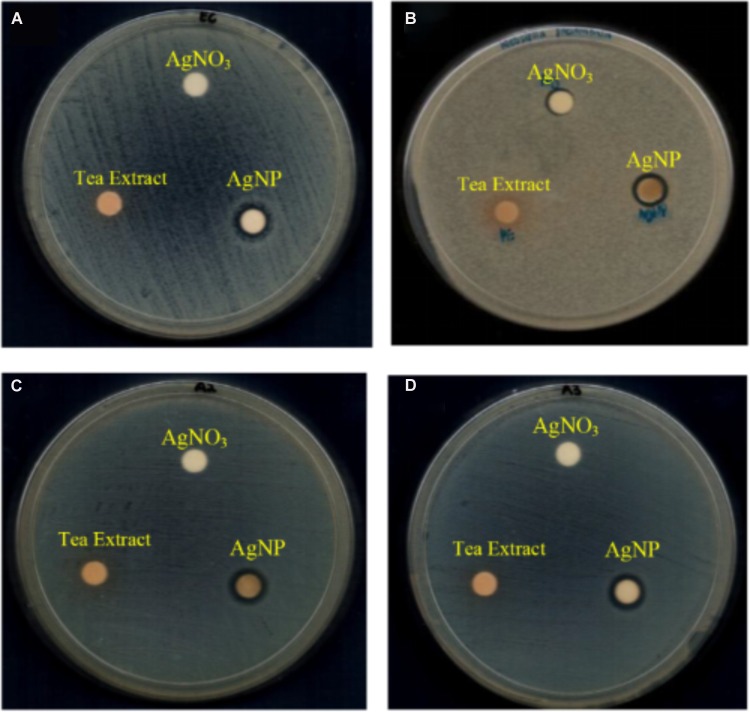
Visible clear zone produced by tea leaves extract mediated AgNP against four species of foodborne pathogens: **(A)** *E. coli* ATCC 25922, **(B)** *K. pneumoniae* ATCC 13773, **(C)** *S.* Typhimurium ATCC 14028, and **(D)** *S.* Enteritidis ATCC 13076.

The antibacterial activity of AgNPs was determined against four species of Gram-negative foodborne pathogens: *E. coli* ATCC 25922, *K. pneumoniae* ATCC 13773, *S.* Typhimurium ATCC 14028, and *S*. Enteritidis ATCC 13076. The results for disk diffusion test, MIC and MBC of the AgNPs are summarized in **Table 1**. For the disk diffusion test, the presence of clear zone around the AgNPs disk suggesting that the AgNPs possessed antibacterial activity which is able to inhibit the growth of the Gram-negative foodborne pathogens. As previous study by Guzman et al. (2012), reported that AgNPs employed antibacterial activity on Gram-negative bacteria. The visible clear zone produced by AgNPs against four different species of Gram-negative bacteria is showed in **Figure 1**.

**Table 1:** The diameter of zone inhibition (mm), MIC value (μg/mL), and MBC value (μg/mL).

Bacteria	Diameter of inhibition zone (mm)	MIC (μg/mL)	MBC (μg/mL)
*Escherichia coli*	15	7.8	7.8
*Klebsiella pneumoniae*	10	3.9	3.9
*Salmonella* Typhimurium	20	3.9	7.8
*Salmonella* Enteritidis	20	3.9	3.9


Disk diffusion test was described as the preliminary study in screening the antibacterial activity of an antimicrobial agent; therefore, a further evaluation in determining the antibacterial activity of AgNPs using MIC value was needed (Burt, 2004). MIC was defined as the lowest concentration of the antibacterial agent to inhibit the growth of bacteria by serial dilution. As showed in **Table 1**, the MIC values of AgNPs against the foodborne pathogens were ranged from 3.9 to 7.8 μg/mL. *K. pneumonia*, *S.* Typhimurium and *S.* Enteritidis showed the MIC value of 3.9 μg/mL while *E. coli* showed the MIC value of 7.8 μg/mL. MBC is the lowest concentration of antibacterial agent to kill the bacteria (showed no growth on the agar plate). In the study, MBC for *K. pneumoniae* and *S.* Enteritidis were 3.9 μg/mL while *S.* Typhimurium and *E. coli* showed the MBC value of 7.8 μg/mL. The MIC and MBC value of *E. coli* showed that *E. coli* was less susceptible to AgNPs. This may due to the positive charges of AgNPs trapped and blocked by lipopolysaccharide, thus make *E. coli* less susceptible to AgNPs (Lara et al., 2010b).

Resazurin dye was used in the study as an indicator in the determination of cell growth, especially in cytotoxicity assays (McNicholl et al., 2007). Oxidoreductases within viable cells reduced the resazurin salt to resorufin and changed the color from blue non-fluorescent to pink and fluorescent. According to McNicholl et al. (2007), resazurin dye has been applied for decades to check for the bacterial and yeast contamination in milk.

**FIGURE 2:**
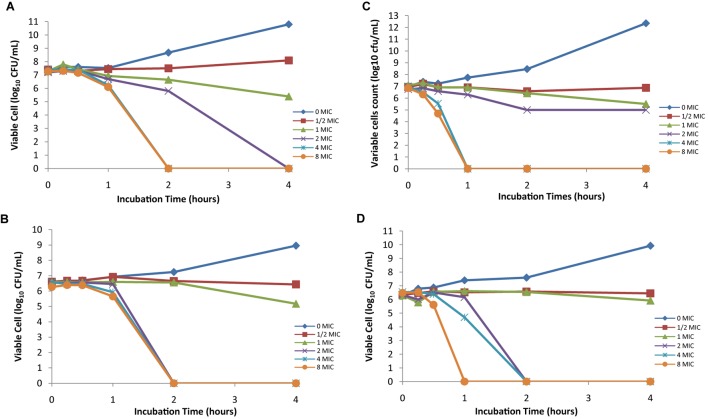
Time-kill plots of AgNPs against **(A)** *E. coli* ATCC 25922, **(B)** *K. pneumoniae* ATCC 13773, **(C)** *S.* Typhimurium ATCC 14028, and **(D)** *S.* Enteritidis ATCC 13076 at different concentration and time-length.

The time kill activity of four foodborne pathogens is shown in **Figure 2**. The bactericidal activity of AgNPs is effective against the selected Gram-negative pathogens; the reduction in the number of CFU/mL was ≥3 Log units (99%). The bactericidal endpoint of AgNPs for *E. coli* was reached after 2 h of incubation at 4× MIC (31.2 μg/mL) and 8× MIC (62.4 μg/mL); while for *K. pneumoniae*, the bacteria was killed after 2 h of incubation at 2× MIC (7.8 μg/mL), 4× MIC (15.6 μg/mL), and 8× MIC (31.2 μg/mL). *S.* Typhimurium was killed after 1 h of incubation at 4× MIC (15.6 μg/mL) and 8× MIC (31.2 μg/mL). The bactericidal endpoint of AgNP for *S.* Enteritidis was reached after 2 h of incubation at 2× MIC (7.8 μg/mL) and 4× MIC (15.6 μg/mL); however, the end point reached faster after 1 h of incubation at 8× MIC (31.2 μg/mL). No significant differences (*P* > 0.05) were found among the tested Gram-negative foodborne pathogens. This indicates that AgNPs are broad spectrum antimicrobial agents which exert the same effect to all Gram-negative bacteria strains.

Silver nanoparticles are well-known as the most universal antimicrobial substances due to their strong biocidal effect against microorganisms, which has been used for over the past decades to prevent and treat various diseases (Oei et al., 2012). AgNPs are also widely used as anti-fungal (Kim et al., 2009), anti-inflammatory (Nadworny et al., 2010), and anti-viral properties (Lara et al., 2010a). Recently, non-hazardous AgNPs can easily be synthesized using a cost-effective method and tested as a new type of antimicrobial agents.

In this study, the application of AgNPs as an antimicrobial agent was tested against selected Gram-negative bacteria on agar plate and liquid medium. The results showed that the tested bacteria could completely inhibit by AgNPs. The inhibition of bacteria growth was reported affected by the concentration of AgNPs and bacteria used in the experiments (Sondi and Salopek-Sondi, 2004). The green synthesized AgNPs in this study are able to inhibit the high concentration of bacteria (approximately 10^6^ CFU/mL). This indicated that AgNPs showed an excellent antimicrobial effect as the high CFU concentration of bacteria used in this study are rarely appeared in real-life systems.

The antibacterial activity of AgNPs has been reported by many researchers. However, the MIC values from the previous studies showed the range through a large extent of variation. Therefore, the comparison of the results is difficult as there is no standard method for determination of antibacterial activity of AgNPs and different methods have been applied by the researchers (Zarei et al., 2014). In this study, AgNPs exhibit a good antibacterial activity against the tested foodborne pathogens. Based on the results, the tested bacteria were able to kill in a shorter time at low concentration of AgNPs. This may due to the cell wall structure of Gram-negative bacteria. The characteristic cell wall structure of Gram-negative bacteria is different from Gram-positive bacteria. Gram-negative bacteria have a cytoplasmic membrane, a thin peptidoglycan layer, and an outer membrane containing lipopolysaccharide. There is a space between the cytoplasmic membrane and the outer membrane called the periplasmic space or periplasm. The periplasmic space contains the loose network of peptidoglycan chains known as the peptidoglycan layer.

The rapid reproduction time of bacteria is one of the main causes of bacteria’s infectivity (Lara et al., 2010b). However, the reproduction time of the bacteria could be an ideal way to prevent the viable infection as AgNPs were effective in inhibiting and killing the bacteria in a dose and time dependent manner as shown in the time-kill assays. Zhang et al. (2016) reported that the smaller size of AgNPs could cause more toxicity to the bacteria and show better bactericidal effect compared to the larger particles as they have larger surface area. Previous study by Agnihotri et al. (2014) found that the antibacterial efficacy was increased for the AgNPs with less than 10 nm size. They also concluded that AgNPs with the size of 5 nm have the fastest antibacterial activity compared to others size of AgNPs.

Silver nanoparticles have emerged as antimicrobial agents against multidrug resistant bacteria due to their high surface-area-to-volume ration and unique chemical and physical properties. AgNPs have particle size ranging from 1 to 100 nm. The surface area-to-volume ratio of AgNPs increases as the particles size decreases. Morones et al. (2005) reported that AgNPs with the size of 10-100 nm showed strong antimicrobial effect against both Gram-positive and negative bacteria. The small particle size enables AgNPs to adhere to the cell wall and penetrate into the bacteria cell easily, which in turn improves their antimicrobial activity against bacteria. The antimicrobial effects of AgNPs against multidrug resistant bacteria have been studied by many researchers and it was proved that AgNPs are effective against multidrug resistant bacteria such as multidrug resistant *E. coli* (Paredes et al., 2014; Kar et al., 2016), multidrug resistant strain of *Pseudomonas aeruginosa* (Durairaj et al., 2012), methicillin-resistant *Staphylococcus aureus* (MRSA) (Paredes et al., 2014; Yuan et al., 2017), and extended-spectrum β-lactam (ESBL) producing bacteria (Doudi et al., 2013; Subashini et al., 2014).

On the other hands, AgNPs are advantageous compared to conventional chemical antimicrobial agents as the major problem caused by the conventional chemical antimicrobial agents is multidrug resistance. The effectiveness of the chemical antimicrobial agents depends on the specific binding of the microorganisms with the surface and metabolites of the antimicrobial agents. However, the chemical antimicrobial agents are limited to use especially in medical field as various microorganisms have developed multiple resistance traits over a period of generations. Thus, the development of AgNPs could be an alternative way to overcome the multidrug resistance microorganisms as bacteria are less likely to develop resistance to metal nanoparticles compared to the conventional antibiotics.

The exact mechanisms of AgNPs against bacteria still remain unknown. However, there are some researchers proposed that the action of AgNPs on bacteria may due to its ability to penetrate into the cell (Sondi and Salopek-Sondi, 2004), the formation of free radicals (Danilczuk et al., 2006; Kim et al., 2007), the inactivation of proteins in the cell by silver ions (Rai et al., 2012) and the production of reactive oxygen species (ROS) (Dakal et al., 2016). Besides that, there are also some factors in affecting the bactericidal mechanisms of AgNPs such as the concentration of AgNPs and bacteria class (Kim et al., 2007; Zhang et al., 2014), shape (Pal et al., 2007; Meire et al., 2012), size (Martinez-Castanon et al., 2008), and the combination of various antibiotics (Fayaz et al., 2010; Singh et al., 2013).

## Conclusion

Silver nanoparticles showed significant antibacterial activity against the selected Gram-negative foodborne pathogens. Thus, AgNPs might be a good alternative to develop as antibacterial agent against the multidrug-resistant strains of bacteria. The applications of AgNPs may lead to valuable findings in various fields such as medical devices and antimicrobial systems.

## Author Contributions

YL, BC, YR, and RS developed the study design. YL and CK carried out the confirmation for the selected foodborne pathogens. MN provided culture media and technical advice in the study. YL interpreted the data, drafted the manuscript, and revised the manuscript. YR, M-A-RN-K, CK, BC, and RS checked on the manuscript. All authors read and approved the final version of the manuscript.

## Conflict of Interest Statement

The authors declare that the research was conducted in the absence of any commercial or financial relationships that could be construed as a potential conflict of interest.
